# Time-resolved FRET reports FGFR1 dimerization and formation of a complex with its effector PLCγ1

**DOI:** 10.1016/j.jbior.2015.09.002

**Published:** 2016-01

**Authors:** Louis Perdios, Tom D. Bunney, Sean C. Warren, Christopher Dunsby, Paul M.W. French, Edward W. Tate, Matilda Katan

**Affiliations:** a Institute of Structural and Molecular Biology, Division of Biosciences, University College London, Gower St, London WC1E 6BT, UK; b Department of Chemistry, Imperial College London, South Kensington Campus, Exhibition Road, London SW7 2AZ, UK; c Department of Physics, Imperial College London, South Kensington Campus, Exhibition Road, London SW7 2AZ, UK

**Keywords:** Fibroblast growth factor receptor, Phospholipase Cγ, Time-resolved FRET

## Abstract

*In vitro* and *in vivo* imaging of protein tyrosine kinase activity requires minimally invasive, molecularly precise optical probes to provide spatiotemporal mechanistic information of dimerization and complex formation with downstream effectors. We present here a construct with genetically encoded, site-specifically incorporated, bioorthogonal reporter that can be selectively labelled with exogenous fluorogenic probes to monitor the structure and function of fibroblast growth factor receptor (FGFR). GyrB.FGFR1KD.TC contains a coumermycin-induced artificial dimerizer (GyrB), FGFR1 kinase domain (KD) and a tetracysteine (TC) motif that enables fluorescent labelling with biarsenical dyes FlAsH-EDT_2_ and ReAsH-EDT_2_. We generated bimolecular system for time-resolved FRET (TR-FRET) studies, which pairs FlAsH-tagged GyrB.FGFR1KD.TC and N-terminal Src homology 2 (nSH2) domain of phospholipase Cγ (PLCγ), a downstream effector of FGFR1, fused to mTurquoise fluorescent protein (mTFP). We demonstrated phosphorylation-dependent TR-FRET readout of complex formation between mTFP.nSH2 and GyrB.FGFR1KD.TC. By further application of TR-FRET, we also demonstrated formation of the GyrB.FGFR1KD.TC homodimer by coumermycin-induced dimerization. Herein, we present a spectroscopic FRET approach to facilitate and propagate studies that would provide structural and functional insights for FGFR and other tyrosine kinases.

## 1 Introduction

Fibroblast growth factors (FGFs) and their receptors (FGFR1-4) play a critical role in many physiological processes including embryogenesis, wound healing, inflammation and angiogenesis as well as adult tissue homeostasis (Beenken and Mohammadi, 2009). Importantly, aberrant FGFs/FGFRs signalling has been causatively linked to several developmental syndromes and a broad range of human malignancies (Carter et al., 2015, Helsten et al., 2015). This involvement in the pathology of many cancer types provided a strong rationale for development of effective agents for these targets; consequently, there is a large ongoing effort to develop FGFR inhibitors as anticancer treatments (Touat et al., 2015).

FGFs mediate their diverse biological responses by binding to FGFRs, leading to their dimerization and autophosphorylation; despite extensive insights into isolated extracellular and intracellular regions of FGFRs, overall process of dimerization is not clearly defined (Goetz and Mohammadi, 2013, Maruyama, 2014). Subsequent to dimerization, similar to other receptor tyrosine kinases (RTKs), activated FGFRs dimers recruit and phosphorylate a complement of signalling molecules that mediate the cellular activities of FGFs (Bradshaw et al., 2015, Lemmon and Schlessinger, 2010, McCubrey et al., 2015). In case of FGFRs, the two main direct binding proteins that propagate signalling are the adaptor molecule FRS2 and phospholipase Cγ (PLCγ) (Beenken and Mohammadi, 2009, Eswarakumar et al., 2005). PLCγ is recognized as a key component downstream of RTKs involved in dynamic coupling of modifying enzymes with signalling lipids (Blind, 2014, Follo et al., 2015) and, furthermore, has been recently implicated in disease development through gain-of-function mutations (Koss et al., 2014).

Several lines of experimental evidence have demonstrated that FGFR is constitutively bound to FRS2 via FGFR juxtamembrane region, whereas PLCγ is recruited to the C-terminal tail of the receptor upon autophosphorylation of a highly conserved tyrosine (Y766 in case of FGFR1) (Eswarakumar et al., 2005). The main interacting domain in PLCγ is the N-terminal Src-homology 2 domain (nSH2) present within the PLCγ regulatory region (or γ-specific array, γSA); the interaction involves not only the canonical binding site centered on pY766 at the FGFR1 tale, but also a secondary binding site (Bae et al., 2009). Interestingly, the selectivity of PLCγ binding and signalling via activated FGFR1 are determined by interactions between this secondary binding site on nSH2 domain and a region in FGFR1 kinase domain. Thus, binding of nSH2 from PLCγ1 to two distinct sites on FGFR1 (the tale and kinase domain) represents a highly specific and high affinity (kD about 5 nM) event that is, overall, dependent on activation status of FGFR (Bae et al., 2009, Bunney et al., 2012). Measurements of this interaction using fluorescent reporters and FRET could provide a sensitive method to assess activation of FGFR *in vitro* and in cells or an inhibition of this process in the presence of small molecule drugs.

We here describe generation and characterisation of constructs that provide new tools to assess spatiotemporal mechanistic details of dimerization and downstream signalling. We generated GyrB.FGFR1KD.TC construct comprising a coumermycin-induced artificial dimerizer (GyrB), FGFR1 kinase domain (KD) – exhibiting wild-type analogous autophosphorylation, substrate recruitment, and inhibitor response in kinase assays and chromatography measurements – and a tetracysteine (TC) motif that enables fluorescent labelling with biarsenicals FlAsH-EDT_2_ and ReAsH-EDT_2_. We conceived systems for TR-FRET studies, which pair either FlAsH- and ReAsH-tagged GyrB.FGFR1KD.TC or pair FlAsH-tagged GyrB.FGFR1KD.TC and nSH2 domain of PLCγ fused to mTurquoise fluorescent protein (mTFP). We demonstrated coumermycin-induced dimerization and phosphorylation-dependent TR-FRET readout of complex formation between mTFP.nSH2 and GyrB.FGFR1KD.TC.

## 2 Material and methods

### 2.1 TR-FRET

For fluorescence lifetime decay measurements in a quartz cuvette using a TR-FRET multidimensional spectrofluorometer. GyrB.FGFR1KD.TC (1 μM) tagged with FlAsH-EDT_2_ (5 μM and 50 μM) and its partner mTFP.nSH2 (1 μM) in 40 mM Tris–HCl (pH8.0), 20 mM MgCl_2_, 20 mM NaCl, 100 μM TCEP, and 100 μM Na_3_VO_4_ were incubated in the presence or absence of ATP (50 μM) in at ambient conditions for 30 min. Excitation of the FRET pair was performed at 410–425 nm using the supercontinuum source, and fluorescence emission detected at 430–520 nm (10 nm steps) in order to record the decrease in fluorescence lifetime of the donor mTFP. Analysis of lifetime and anisotropy decays was performed using TRFA data processor (Scientific Software Technologies Center, Minsk, Belarus).

Details for cloning, expression and purification of recombinant proteins, kinase assays, *in vitro* labelling of purified proteins and Supplementary Figures can be found in Supplementary Information.

## 3 Results

### 3.1 Constructs for a TR-FRET approach to detect FGFR1 dimerization and complex formation with PLCγ

**Fig. 1:**
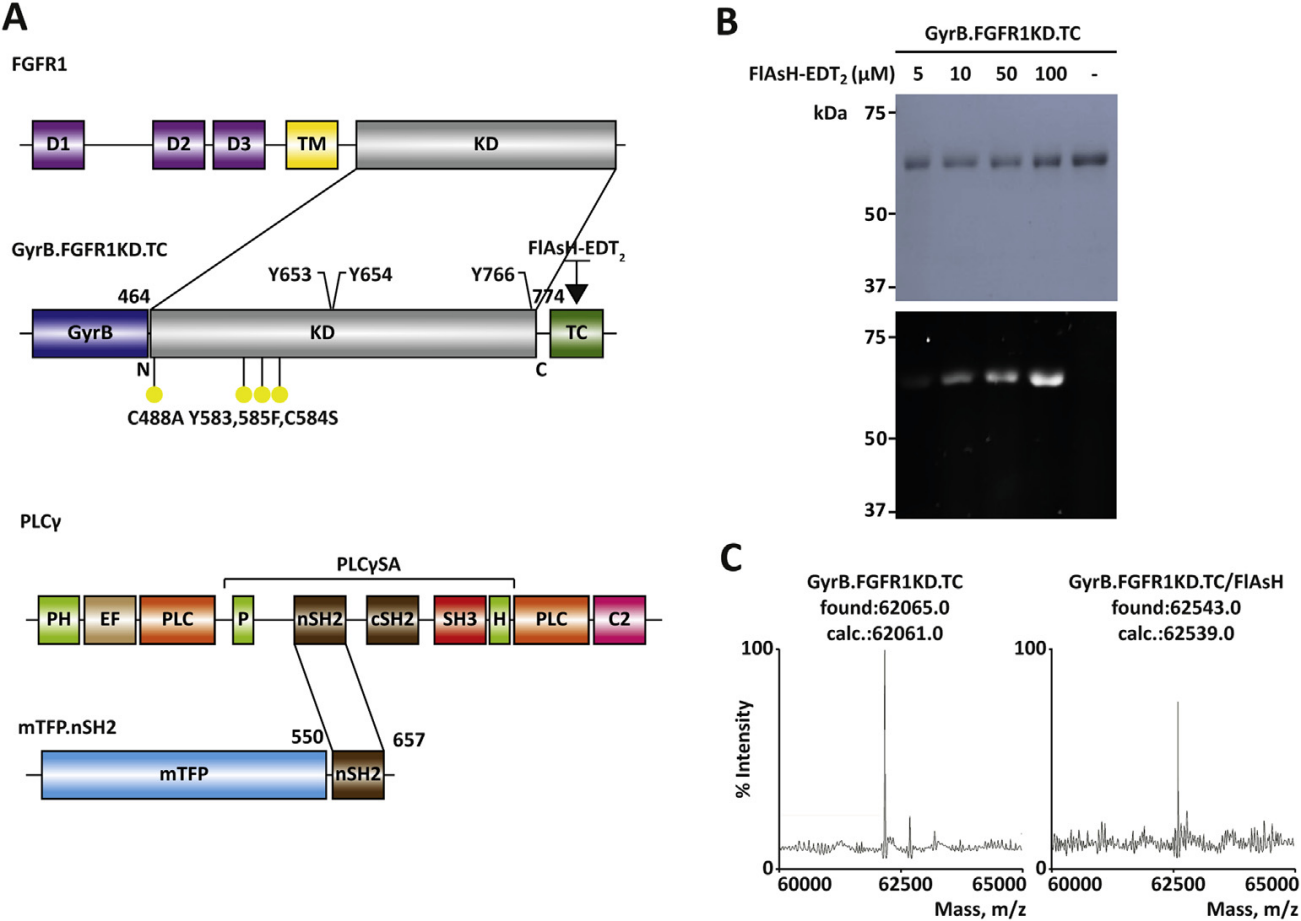
Representation of the constructs used in dimerization and complex formation of FGFR1KD. (A) Representation of the wild type FGFR1 and PLCγ as well as domains of protein constructs used in this study. FGFR1 (top) comprises Ig-like domains 1, 2, and 3 (D1, 2, and 3, respectively), trans-membrane domain (TM) and intracellular kinase domain (KD). KD (with mutations shown as yellow dots) is incorporated in GyrB.FGFR1KD.TC that also includes an N-terminal GyrB and a C-terminal TC motif. PLCγ (bottom) contains domains common to PLC family: N-terminal pleckstrin homology domain (PH), EF–hands (EF), catalytic domain (PLC, X-box and Y-box, shown as two parts) and C2 calcium/lipid-binding domain (C2). PLCγ Specific Array (PLCγ SA) comprises a split PH domain (PH), two SH2 domains (nSH2 and cSH2) and the SH3 domain. mTurquoise.nSH2 fusion protein (mTFP.nSH2) comprises an mTurquoise (mTFP) and an nSH2 domain. (B) Specific and quantitative labelling of GyrB.FGFR1KD.TC upon incubation with FlAsH-EDT_2_ demonstrated by SDS-PAGE (Coomassie staining) (top) and in-gel fluorescence (bottom). (C) Mass spectrum of the full length intact GyrB.FGFR1KD.TC before (left) and after (right) labelling with FlAsH-EDT_2_. (For interpretation of the references to colour in this figure legend, the reader is referred to the web version of this article.)

Accumulating evidence show that ligand-induced dimerization of RTK extracellular regions leads to activation of the intracellular KD, which, in turn, alters complex downstream signalling networks (Lemmon and Schlessinger, 2010). However, intact RTKs are difficult to generate and reconstruct into *in vitro* signalling systems. In order to study the molecular interactions of the FGFR1 *in vitro*, a dimerizer was incorporated at the N-terminus and a fluorescent self-labelling peptide sequence at the C-terminus of FGFR1KD (Fig. 1 A, top). Our previous work (Bunney et al., 2012, Bunney et al., 2015) involved the construction of a synthetic open reading frame (ORF) encoding for a mutant form of human FGFR1 kinase domain (KD), FGFR1KD [amino acids 457–774, (Y463, 583, 585F), (L457V, C488A, C584S)] that forms a central part of our new construct (Fig. 1 A, top). FGFR1 dimerization could be monitored *in vitro* by using an artificial dimerization motif at the N-terminus of the KD to mimic the extracellular portion of the wild-type protein. For this purpose, the N-terminal 24-kDa subdomain of the B subunit of bacterial DNA gyrase (GyrB), which dimerizes in the presence of the antibiotic coumermycin (Farrar et al., 1996, Liu et al., 2003), was incorporated into the construct, generating GyrB.FGFR1KD. Using recombinant cloning methods, an optimised TC motif (FLNCCPGCCMEP) (Spagnuolo et al., 2006) was introduced at the C-terminus of GyrB.FGFR1KD to generate GyrB.FGFR1KD.TC (Fig. 1A, top). The protein was then isolated from transformed *Escherichia coli* strain C41(DE3). The optimised FlAsH- or ReAsH- binding TC motif has been previously used to monitor protein structure *in vitro* and in mammalian cells (Luedtke et al., 2007, Martin et al., 2005, Roberti et al., 2007, Spille et al., 2011).

The high affinity of the TC motif to the biarsenical dye (Adams and Tsien, 2008) suggests that the complex should be stable under typical denaturing conditions. The identity and integrity of TC-tagged protein was verified by survival of fluorescence after SDS-PAGE. The recombinant protein was incubated with FlAsH-EDT_2_ for 30 min at room temperature in a modified sample buffer for SDS-PAGE in which the typical thiol reductant (2-mercaptoethanol) was substituted by tris(2-carboxy-ethyl)phosphine (TCEP). After electrophoresis, a single major fluorescent species running at the anticipated molecular weight for the GyrB.FGFR1KD.TC (60.1 kDa) was visible by UV illumination (Fig. 1B). Excess FlAsH-EDT_2_, which could result in increased levels of background fluorescence, is removed as it migrates with the dye front. Subsequent staining with Coomassie Blue confirmed the specific binding of dye to the TC-containing protein. This result suggests that GyrB.FGFR1KD.TC tagged with FlAsH-EDT_2_ (or ReAsH-EDT_2_) can be studied in a mixture of proteins or in a crude lysate. The identity of the fully labelled protein was further confirmed by native mass spectroscopy (Fig. 1C), in which the GyrB.FGFR1KD.TC/FlAsH-EDT_2_ complex was clearly observed as a single peak at the anticipated molecular weight (found: 62543.0, calc.: 62539.0). Based on these data, labelling of GyrB.FGFR1KD.TC is suitable for further FRET experiments to test dimerization.

As a second model system for RT-FRET experiments, the FGFR1 KD and its downstream target PLCγ were used; it has been previously established that a stable complex is formed between the C-terminus of activated FGFR1KD and two different sites on the surface of the nSH2 from PLCγ regulatory region (Fig. 1A, bottom) (Bae et al., 2009). The canonical phosphorylation-dependent binding site consists of a positively charged patch on the surface of the nSH2 domain that binds to pY766 and a hydrophobic pocket in the FGFR1 C-terminal tail. A second binding site of the nSH2 forms mediates high affinity binding by forming contacts with the backside of the C-lobe of the KD away from the activation segment of FGFR1. The co-crystal structure of nSH2 of PLCγ with activated FGFR1KD (PDB code: 3GQI) (Bae et al., 2009), estimates a distance of 28.7 Å between nSH2 (N-terminus) and FGFR1KD (C-terminus), making the protein interaction measurable by FRET. As a FRET donor, cyan fluorescent protein (CFP) variant mTurquoise (mTFP) (excitation: 434 nm, emission: 474 nm) fused to the nSH2 domain of PLCγ (mTFP.nSH2) was used (Fig. 1A, bottom).

### 3.2 Kinase profiling of GyrB.FGFR1KD.TC demonstrates that its biochemical properties match those of the wild-type protein *in vitro*

To determine the *in vitro* tyrosine kinase activity of recombinant GyrB.FGFR1KD.TC, a bioluminescent, ADP detection assay was used (Zegzouti et al., 2009). The kinase substrates used here were PolyE_4_Y_1_ and a part of PLCγ regulatory region, which comprises the two SH2 domains of PLCγ followed by a segment containing Y783 (Fig. 1A, bottom). Inhibitors used here were PD173074, an established ATP-competitive inhibitor of FGFR1 (inhibitor constant, K_i_: ∼44 nM, IC_50_: ∼22 nM) (Mohammadi et al., 1998) and AZD4547, a selective inhibitor of FGFR1 currently in clinical trials (K_i_: 6.1 nM, IC_50_: 0.2 nM) (Bunney et al., 2015, Gavine et al., 2012).

**Fig. 2:**
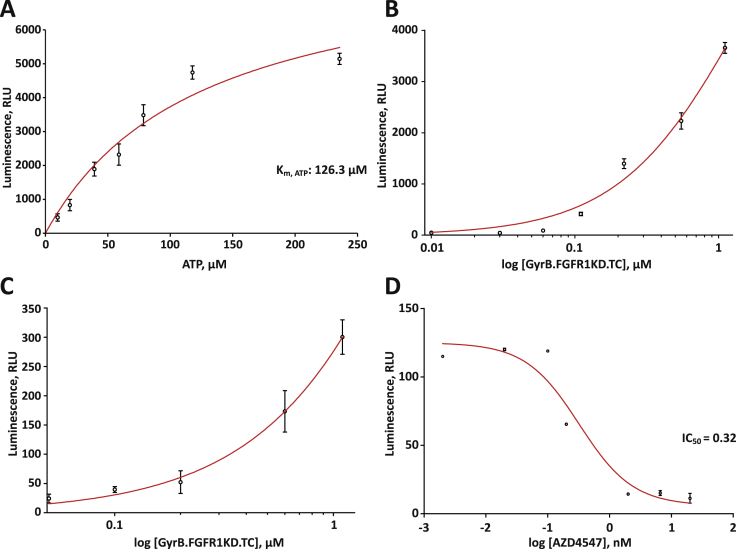
Kinase profiling of GyrB.FGFR1KD.TC by bioluminescent ADP detection assays. (A) Determination of ATP Km for GyrB.FGFR1KD.TC by titrating with 10–240 μM ATP and 0.2 μg/μl PolyE_4_Y_1_. (B) Examination of the dependence of signal linearity to kinase concentration by titration of 0–1.1 μM GyrB.FGFR1KD.TC with 150 μM ATP and 0.2 μg/μl PolyE_4_Y_1_. (C) Autophosphorylation assay at varying concentrations of 0–1.1 μM enzyme and 150 μM ATP. (D) Inhibitor dose response titration of GyrB.FGFR1KD.TC in the presence of 150 μM ATP, 0.2 μg/μl PolyE_4_Y_1_ peptide and inhibitor AZD4547 at varying concentrations of 0.002–20 nM (FGFR1 IC_50_ = 0.2 nM). Kinase inhibition was measured by detecting the decrease in phosphorylation of PolyE_4_Y_1_ after 1 h. Maximal activity of GyrB.FGFR1KD.TC and the background signal were measured in the absence of inhibitor AZD4547. GyrB.FGFR1KD.TC activity was expressed by calculating the ratio between the background-corrected assay signals in the absence and presence of the indicated inhibitor concentrations. Curve fitting for inhibitor dose response titration was performed using GraphPad Prism^®^ sigmoidal dose–response software. In all panels error bars indicate the standard deviations (SDs) of three replicates.

To optimise assay conditions, we first determined the ATP concentration that equals the K_m_ value for GyrB.FGFR1KD.TC (Fig. 2A). The data were fitted to the Michaelis–Menten equation, thereby determining the GyrB.FGFR1KD.TC ATP K_m_ to be 126 μM. After identifying appropriate substrates and an optimal ATP concentration, the dependence of signal linearity to kinase concentration was established in a series of GyrB.FGFR1KD.TC titrations with PolyE_4_Y_1_ (Fig. 2B) and PLCγ SA WT (Supplementary Figure S3A). Here, we have chosen to use 0.3 nM and 30 nM of GyrB.FGFR1KD.TC with inhibitors AZD4547 and PD173074 respectively, to guarantee sufficiently high assay signal as well as substrate conversion below 70%. In the last step of the optimisation process, the contribution of the autophosphorylation state of the kinase on the total assay signal was examined in the absence of substrate. Increasing enzyme concentration were titrated with 150 μM ATP (Fig. 2C), resulting in higher levels of signal readout corresponding to autophosphorylation.

In order to validate the optimised GyrB.FGFR1KD.TC assay, the kinase activity was quantified with increasing concentrations of inhibitor AZD4547 (Fig. 2D) and reference inhibitor PD173074 (Supplementary Figure S3B). For AZD4547, the IC_50_ value of 0.32 nM was in good agreement with the published value of 0.20 nM. For PD173074, the ${\text{IC}}_{50}^{\text{obs}}$ was 13 nM, an estimation that diverges slightly from the reported value of ∼22 nM. This may be ascribed to dissimilarities between the assay format used here and the one in literature, where a radiometric readout assay format and full length FGFR1 were used to measure the IC_50_ values.

### 3.3 Coumermycin-induced dimerization of FlAsH and ReAsH labelled GyrB.FGFR1KD.TC proteins results in a FRET readout

Extracellular dimerization of FGFRs involves the formation of 2:2:2 FGF:FGFR:heparin ternary complex. The recombinant proteins used in this study contain the intracellular KD activity domain, which is suitable for enzyme activity assays, but do not contain the extracellular domain of FGFR1. Attempts to get multi-domain FGFR constructs were not pursued as they show low expression levels in *E. coli*, due to inadequate solubility and misfolding.

To achieve artificial dimerization of GyrB.FGFR1KD.TC, a model involving FGF ligand-mimic coumermycin, that induces dimerization of GyrB, was used. Coumermycin binds GyrB at a stoichiometric ratio 1:2, whereas another antibody, novobiocin, binds GyrB at 1:1 ratio (Ali et al., 1993) and was thereby suitable as a control of obtaining exclusively monomeric protein population. To first test this dimerization model biochemically, analytical size-exclusion chromatography (SEC) was used before and following incubation with antibiotics. Samples incubated with coumermycin (GyrB to antibiotic molar ratio, 1.5:1) exhibited major peaks that corresponded to the presence of predominantly the dimer (Supplementary Figure S4B). The retention time of the coumermycin-bound dimer was equivalent to the retention time of species of 120 kDa when compared to SEC standard molecular weight markers (Supplementary Figure S1A).

**Fig. 3:**
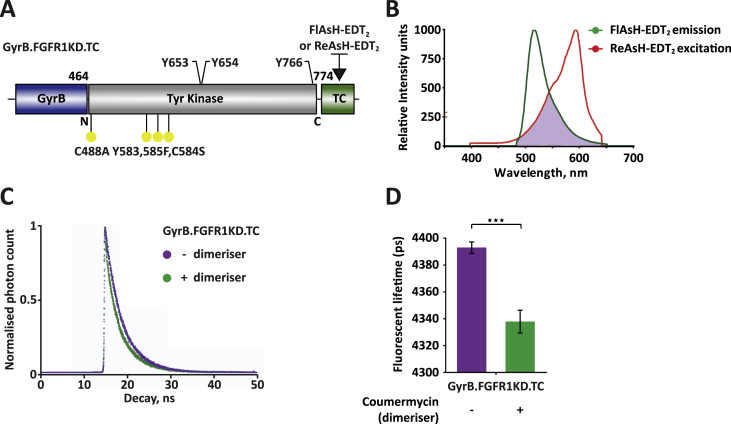
Coumermycin-induced dimerization of GyrB.FGFR1KD.TC observed by FRET. (A) Representation of the construct used for the TR-FRET investigating coumermycin-induced dimerization. (B) Spectral overlap as precondition for FRET. The spectral overlap integral is indicated by the purple area. The region of overlap between the excited donor's emission spectrum (green line) and the acceptor's absorption spectrum (orange line) has to be significant for FRET to occur. (C) Single exponential fit of the fluorescence decay (normalised photon count) over time from fluorescence lifetime decay measurements in a quartz cuvette using a TR-FRET multidimensional spectrofluorometer. (D) Donor lifetime decrease upon dimerization showing statistical significance assessed using data from a minimum of three repeats by a two-tailed unpaired t-test (***0.0001 < *P* < 0.001). (For interpretation of the references to colour in this figure legend, the reader is referred to the web version of this article.)

Coumermycin-induced dimerization was subsequently investigated by TR-FRET. In these studies FlAsH-EDT_2_ is used as the donor to fluorescently tag a population of GyrB.FGFR1KD.TC while red-emitting biarsenical ReAsH-EDT_2_ (excitation: 593 nm, emission: 608 nm), which binds to the C-terminal TC motif of GyrB.FGFR1KD.TC (Supplementary Figure S2), is issued as the acceptor to tag another population of the same protein (Fig. 3A). The suitability of FlAsH-EDT_2_ and ReAsH-EDT_2_ as a donor acceptor pair was demonstrated *in vitro* by bulk spectrofluorimetry (Fig. 3B), which highlighted that the two dyes have ideal spectral overlap for FRET to occur. The change in donor emission in the presence and absence of coumermycin was analysed by fitting the decays using single exponential models (Fig. 3C). Addition of coumermycin to the mixture of proteins tagged for FlAsH-EDT_2_ and ReAsH-EDT_2_ resulted in a decrease in lifetime of ∼60 ps for the sample (Fig. 3D). Thus, under conditions of dimerization, the overall dye proximity increases resulting in faster lifetime decay of the donor and FRET. Since this is triggered by addition of coumermycin to the sample in solution, FRET can be attributed to the formation of dimerization complexes between FlAsH-tagged proteins and ReAsH-tagged proteins.

### 3.4 TR-FRET detection of the complex-formation between FlAsH-labelled GyrB.FGFR1KD.TC and mTFP.nSH2

**Fig. 4:**
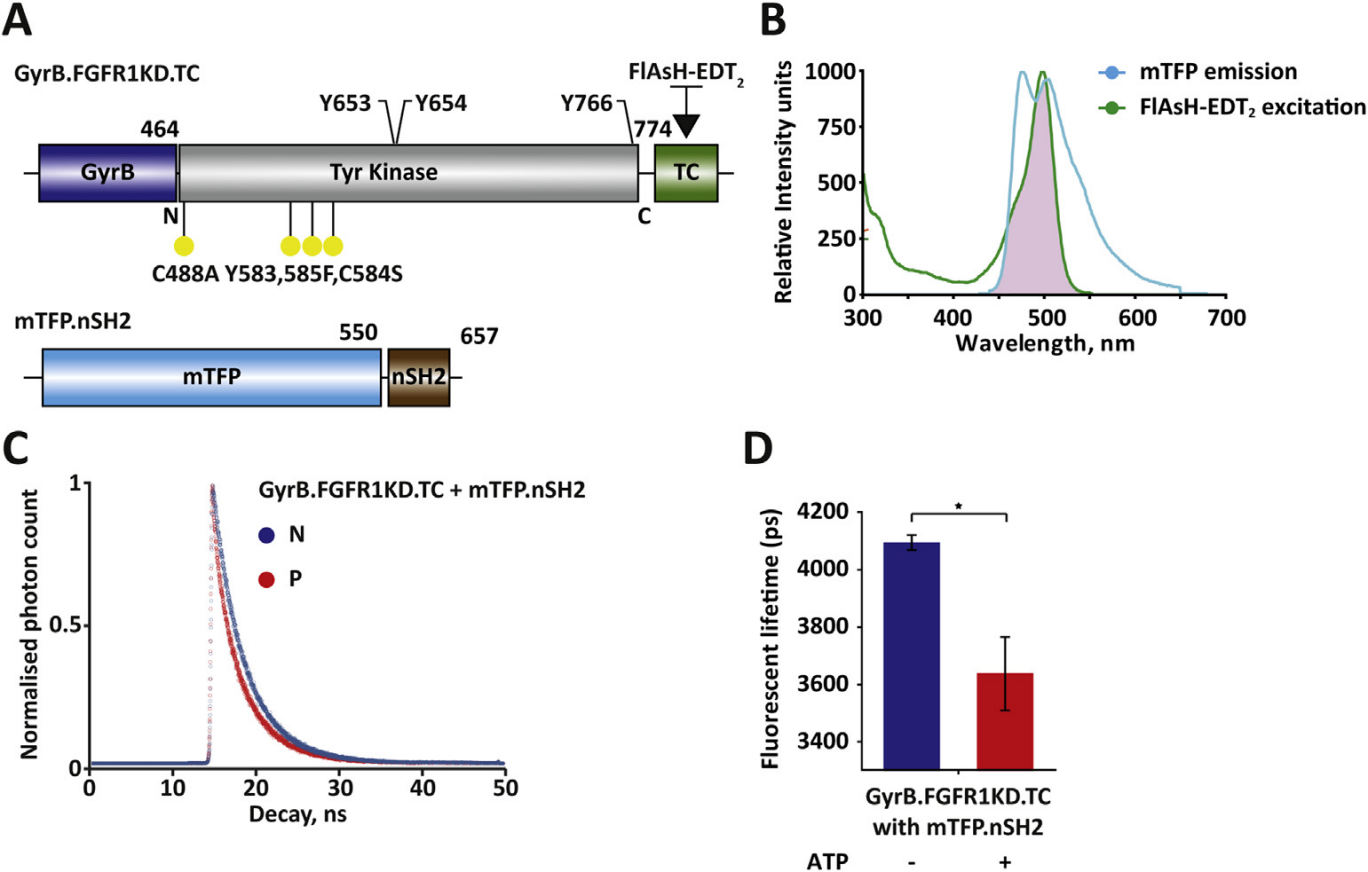
Complex formation between GyrB.FGFR1KD.TC and mTFP.nSH2 observed by FRET. (A) Representation of the constructs used for the TR-FRET examining complex formation. (B) Spectral overlap (purple area) between the excited donor's emission spectrum (green line) and the acceptor's absorption spectrum (orange line). (C) Single exponential fit of the fluorescence decay (normalised photon count) over time from fluorescence lifetime decay measurements. (D) Donor lifetime decrease upon complex formation showing statistical significance assessed using data from a minimum of three repeats by a two-tailed unpaired t-test (*0.0001 < *P* < 0.001). (For interpretation of the references to colour in this figure legend, the reader is referred to the web version of this article.)

For TR-FRET measurements, GyrB.FGFR1KD.TC was reacted with FlAsH-EDT_2_ and then incubated with mTFP.nSH2 for 30 min at room temperature (Fig. 4A). The suitability of FlAsH-EDT_2_ as a donor and mTFP as an acceptor was demonstrated *in vitro* by bulk spectrofluorimetry (Fig. 4B). Next, fluorescence lifetime decays of the samples were recorded in the presence and absence of ATP. To interpret these data, exponential decay models were fitted to the decays; it was apparent that both mTFP.nSH2 by itself and the protein pair were well fit to a single exponential decay (Fig. 4C). Addition of ATP to the incubated protein pair resulted in a large decrease in lifetime of ∼500 ps for the sample (Fig. 4D). The fluorescence decay observed reflects the increase in spatial proximity of the donor and acceptor fluorophores, which was attributed to direct interaction between the nSH2 domain of PLCγ and activated FGFR1KD.

The interaction between GyrB.FGFR1KD.TC and mTFP.nSH2 was also confirmed using SEC; the incubated proteins eluted at the same retention time (elution volume 14.7 mL) indicating formation of a GyrB.FGFR1KD.TC–mTFP.nSH2 complex (Supplementary Figure S4A).

## 4 Discussion

We here demonstrate, using a TR-FRET approach, the suitability of GyrB dimerizer for studies of FGFR signalling and application of bimolecular mTFP/FlAsH.TC system for monitoring the phosphorylation-dependent complex formation between the nSH2 domain of PLCγ and functional GyrB.FGFR1KD.TC.

Use of non-physiological dimerization elements has been widely used to study signal transduction cascades triggered by dimerization (Fegan et al., 2010). This approach has also been applied to FGFR; for example, a construct incorporating FKBPv at the C-terminus of the FGFR KD and dimerization in the presence of a drug (AP20187) provided a basis for specific, inducible and ligand independent activation of FGFR1 signalling in cells and mouse models (Acevedo et al., 2007, Freeman et al., 2003, Tomlinson et al., 2012, Welm et al., 2002). We here demonstrate that GyrB allows not only previously observed dimerization in cells (Liu et al., 2003), but also generation of purified, functional FGFR-GyrB fusion proteins (Fig. 1, Fig. 3) for further structural and *in vitro* studies of elements critical for dimerization and high-throughput screens for disruption of this key activation step.

Interactions between RTKs and SH2 domains of downstream effectors have been explored for their potential to report signalling events in cells and thus provide a platform for drug discovery that would overcome one of the common problems based on kinase assays *in vitro*: the identification of potent hits *in vitro* with poor cellular activity. One recent EGFR biosensor that allows measurements of EGFR clustering as a readout of activation in cells is based on binding of the green fluorescent protein fused to two tandem SH2 domains from adapter protein Grb2; it was subsequently shown that this system provides an important high throughput system for drug discovery performed in a cellular context (Antczak et al., 2012). We here suggest that the highly specific, high affinity interaction between FGFR1 and nSH2 domain from PLCγ (Bae et al., 2009, Bunney et al., 2012) can similarly be used in drug discovery. We generated a FRET pair for TR-FRET detection of the complex-formation between FlAsH-labelled GyrB.FGFR1KD.TC and mTFP.nSH2 (Fig. 4). The decrease in fluorescence lifetime of mTFP depends exclusively on its molecular proximity to the TC motif; hence, FRET occurrence can be attributed to binding of mTFP.nSH2 to activated GyrB.FGFR1KD.TC. The dramatic decrease in fluorescent lifetime of the donor may be ascribed to the high affinity binding of the nSH2 domain to the activated FGFR1KD. *In vitro* experiments with isolated FRET pairs have provided a straightforward readout of the protein–protein interaction and can be adapted for screening of inhibitors that directly affect kinase activity as well as interaction between FGFR and PLCγ. Furthermore, this approach can be developed for cellular FLIM-FRET. Using constructs that ensure membrane localization of GyrB.FGFR1KD.TC and intracellular labelling of this protein in the presence of FlAsH, subsequent activation in response to addition of coumermycin will result in binding of the fluorescent fusion protein mTFP.nSH2 expressed in the same cell and the FRET readout.
