# Supplementary material for: Time-resolved FRET reports FGFR1 dimerization and formation of a complex with its effector PLCγ1
Source: Adv Biol Regul. 2016 Jan;60:6–13. doi: 10.1016/j.jbior.2015.09.002 (PMC4739061; doi:10.1016/j.jbior.2015.09.002)
Supplement: Supplementary file 1 [file mmc1.pdf]

Supplementary material

Table of contents

- Supplementary Experimental Procedures
- Supplementary References
- Supplementary Figure Legends
- Supplementary Figures S1, S2, S3 and S4

Supplementary Experimental Procedures

Expression of recombinant proteins

For expression of His_6.Stag.3C.GyrB.FGFR1KD.TC {which encodes N-terminally hexahistidine tag, S-tag, 3C protease recognition sequence, DNA gyrase B (GyrB) domain, FGFR1KD-3F-0P [457–774, (Y463, 583, 585F) (L457V, C488A, C584S)] and fluorescein arsenical hairpin binder (FlAsH) amino acid sequence (FLN***CC***PG***CC***MEP)} from pTriEx^{TM}4 (Novagen) vector backbone. Constructs were heat-shock transformed into *E.coli* strain C41 (DE3) with pTriEx^{TM}4- His_6.Stag.3C.GyrB.FGFR1KD.TC and pDUET-Cdc37-λPPase (which encodes lambda protein phosphatase (λPPase), an enzyme that hydrolyses phosphorylated tyrosines and Hsp90 co-chaperone Cdc37, which promotes the stability of kinase domains). Cells were recovered in 1 ml of SOC media for 1 h at 37 ºC, and plated on terrific broth (TB) agar plates supplemented with ampicillin (50 µg/mL), spectinomycin (50 µg/mL) and 10 mM glucose. Colonies were inoculated into 500 ml of TB with ampicillin (50 µg/mL) and spectinomycin (50 µg/mL) and grown to an OD600 of 1.0. Cultures were cooled to 15°C and expression was induced with 100 µM IPTG for approximately 16 hours. Cells were harvested by centrifugation and frozen at -80 ºC until processed.

600 µL of Ni^{2+}-NTA beads (Qiagen) were added to the extract and the mixture was incubated with agitation for 1 h at 4 ºC. Beads were collected by centrifugation (10 min, 1000 g). The beads were three times resuspended in 30 mL wash buffer (20 mM Tris-HCl, 30 mM imidazole, 300 mM NaCl, pH 8.0) and spun down at 1000g. Subsequently, the beads were resuspended in 10 mL of wash buffer and transferred to a column. The protein was eluted with 3 ml of wash buffer supplemented with 200 mM imidazole and further purified by size-exclusion chromatography employing a HiLoad 16/60 Superdex 75 Prep Grade column (GE Life Sciences) at a flow rate of 1 mL/min (buffer: 20 mM Tris-HCl, 100 mM NaCl, 2 mM EDTA, pH 7.4). Fractions containing the protein were pooled and concentrated with an Amicon Ultra-15 3 kDa MWCO centrifugal filter device (Millipore). Purified proteins were analyzed by 10% SDS-PAGE and their mass confirmed by mass spectrometry (see Supplementary Information).

Purification of recombinant proteins

For purification of protein domains expressed in C41 (DE3) or BL21 (DE3). Pellets derived from 1 L cultures were thawed on ice and resuspended in 20 ml of chilled lysis buffer (25 mM Tris-HCl, 250 mM NaCl, 40 mM imidazole, 10 mM benzamidine, 100 µg/ml lysozyme, pH 8.0). Resuspension was accomplished by placing the pellets on an orbital shaker at 150 rpm at 4 °C for 30 minutes. A solution of 10% (v/v) Triton-X-100 and 1 K unitz of bovine pancreatic DNAse I was added and the lysate was placed

on the orbital shaker at 150 rpm at 4°C for 1 hour. Cell lysates were clarified by centrifugation at 13,000 rpm at 4°C for 1 hour in an SS34 rotor (Sorvall). The clarified lysate was loaded onto a 5-ml HisTrap (HiTrap Chelating HP, GE Healthcare) on an Akta Explorer system (GE Healthcare) using His Buffer A (25 mM Tris-HCl, 500 mM NaCl, 40 mM Imidazole, 1 mM TCEP, pH 8.0). Non-specific binding of other proteins was removed by washing the column with 10 column volumes of His Buffer A. His-tagged proteins were collected by eluting with linear imidazole gradient from His Buffer A to His Buffer B (25 mM Tris-HCl, 500 mM NaCl, 500 mM imidazole, 1 mM TCEP, pH 8.0) over 5 column volumes. Protein concentrations from the eluent were measured with a Nanodrop (ThermoScientific). The pooled fractions containing the target protein were further incubated with 10 µg of protease per 1 mg of protein (Ulp1 protease for His_6-Sumo, and 3C protease for His_6-3C) overnight at 4°C to cleave of the N-terminal fusion tag. The protein/protease mix was dialysed overnight at 4 °C against 500 ml of Dialysis Buffer (25 mM Tris-HCl, 150 mM NaCl, 10 mM Imidazole and 1 mM TCEP, pH 8.0). Separation of the cleaved tag was achieved by reverse HisTrap chromatography over the 5 ml HisTrap column and material that did not bind was collected. Subsequently, proteins were further purified by strong quaternary ammonium (Q) anion exchange chromatography on a 5 ml HiTrap Q column (GE Healthcare) in Q Buffer A (25 mM Tris-HCl, 20 mM NaCl, 1 mM TCEP, pH 8.0) and eluted with a linear gradient of 10–20 column volumes to 50% of Q Buffer B (25 mM Tris-HCl, 1 M NaCl, 1 mM TCEP, pH 8.0). Fractions containing the target protein were pooled, concentrated in spin concentrators (Vivaproducts) and then further purified by size exclusion chromatography on a Superdex 200 10/300 GL column (GE Healthcare) using Gel Filtration Buffer (25 mM Tris-HCl, 150 mM NaCl, 1 mM TCEP, pH 8.0). Fractions were collected, combined and concentrated in spin concentrators, snap frozen in liquid nitrogen and stored at -80 °C. Purified proteins were analyzed by 10% SDS-PAGE.

Cloning, expression and purification of recombinant proteins

PCR reactions were carried out on G-STORM thermal cycler, followed by digests using DpnI (NEB). The final reaction was transformed into *E.coli* XL10-Gold (Stratagene). Colonies were picked, plasmid prepared (QIAprep Spin Miniprep Kit, Qiagen) and sequenced after manipulation (BigDye3.1, ABI PRISM) to ensure sequence fidelity.

Kinase assays

In vitro kinase assays of recombinant proteins were carried out using ADP-Glo™ Kinase Assay (Promega).The assays were carried out at 25°C in 40 mM Tris-HCl pH 8.0, 20 mM $MgCl_2$, 20 mM NaCl, 100 µM TCEP, and 100 µM Na_3VO_4, in a total volume of 60 µl (15 µl kinase reaction;15 µl ADP-Glo™

Reagent; 30 µl Kinase Detection Reagent) in solid white, flat-bottom 96-well plates. Poly (E_4Y_1) peptide (Sigma-Aldrich) and PLCγSA WT were used as substrates. The latter is an appropriate substrate since during physiological activation of PLCγ by FGFR, the interaction of activated FGFR1 is centred on the nSH2 of PLCγ, while phosphorylation occurs on residue Y783. Tyrosine phosphatase inhibitor sodium orthovanadate was used in the buffer system to preserve the protein tyrosyl phosphorylation state and allow activation of FGFR1KD in the absence of ligand HSPG (Gherzi 1988, Posner 1994). Kinase reactions were stopped after 30 minutes of incubation for enzyme titration and autophosphorylation reactions, and after 60 minutes for inhibitor dose response titrations. ATP-to-ADP standard conversion curves, Z-values and signal-to-background ratio were calculated according to the manufacturer's published procedure in order to assess the linearity of the assay and to calculate the amount of ADP produced during enzymatic titrations. Z' values were calculated for 5 – 100% ATP conversion using 50 µM ATP. For determinations of K_m of the ATP ($K_{m,\ ATP}$) concentration that should be used, reactions contained a peptide concentration of 0.2 µg/µl and ATP at varying concentrations of 10–240 µM. The assay signal in the presence of increasing ATP concentrations was measured and fitted to the Michaelis–Menten equation. The optimal kinase amount was determined when 50% of substrate conversion was achieved (half maximal reaction velocity, $½V_{max}$), and assays were carried out at the apparent $K_{m,\ ATP}$, at 50 µM ATP. Negative control experiments were performed in the absence of ATP, substrate, enzyme and positive controls were performed with FGFR1KD WT (data not shown). Luminescence output was recorded using BMG Labtech FLUOstar Optima plate reader at 520nm. Apparent IC_{50} values were calculated by detecting the decrease in phosphorylation of Poly (E_4Y_1) upon kinase inhibition using AZD4547 and PD173074. Curve fitting for inhibitor dose response titration was performed using GraphPad Prism® sigmoidal dose-response (variable slope) software. Apparent IC_{50} values were then used to measure the approximate inhibitor constant value (K_i) using the Cheng–Prusoff equation, which describes the dependencies between IC_{50} value and ATP concentration for ATP-competitive inhibitors. Error bars indicate the SDs of three replicates.

SDS-PAGE and Western blotting

Sample buffer (4x stock: 62.5 mM Tris.HCl, 2% SDS, 25% glycerol, 0.01% Bromophenol blue, 0.25 M DTT, pH 6.8) was added to protein samples to 1x. Protein denaturation was performed at 100°C for 5 minutes for optimal sample migration. Protein samples were separated by electrophoresis through sodium dodecyl sulphate polyacrylamide gel electrophoresis (SDS-PAGE) and transferred to a polyvinylidene fluoride (PVDF) membrane (equilibrated in methanol) (Millipore) using a standard wet transfer method (buffer: 50 mM Tris-HCl, 380 mM glycine and 20% methanol). For pull-down assays, proteins were visualised by Amido Black staining. For immunoblotting, the membranes were blocked with 5% non-fat

dry milk (Marvel) in TBS pH 7.5 containing 0.1% Tween-20 (Sigma-Aldrich) (TBS-T) for 30-60 min with agitation. Membranes were incubated with the indicated primary antibodies for 1 hour at room temperature or overnight incubation at 4°C. Membranes were washed three times for 5 min in TBS-T followed by incubation with required conjugate diluted in 5% milk/TBS-T for 1 hour with agitation. Blots were subject to three washes in TBS-T and developed using with ECL prime detection kit (Pierce) and exposed to Hyperfilm ECL (Amersham Biosciences).

In-gel fluorescence of GyrB.FGFR1KD.TC with FlAsH-EDT$_2$ or ReAsH-EDT$_2$

FlAsH-EDT$_2$ (10 – 100 µM) or ReAsH-EDT$_2$ (10 – 50 µM) were added to Laemmli sample buffer [4x stock: 62.5 mM Tris.HCl pH 6.8, 2% SDS, 25% glycerol, 0.01% Bromophenol blue, 50 mM TCEP (neutralized with 3.5 equiv of Tris.HCl pH 8.0)]. The protein and sample buffer were kept at room temperature for 15-30 min and loaded onto the SDS-PAGE that was run as usual. The protein-FlAsH complexes were visualized on the unstained or unfixed gel by illumination with either an ultraviolet light-box or by using a CCD camera with appropriate filters for fluorescence of FlAsH (excitation: 511 nm, emission: 527 nm) or ReAsH (excitation: 593 nm, emission: 608 nm).

Native Mass Spectroscopy

Samples were analysed by HPLC, using a Waters Premier *quadrupole time of flight* (Q-Tof) system with a Waters 2795XC Separations Module and Waters Symmetry ® C18 column (2.1x100 mm, 3.5 µm). Mobile phase A (water + 0.1% formic acid) and mobile phase B (acetonitrile + 0.1% formic acid) were used with a flow rate of 600 µL/min, column temperature of 40 ^{o}C and injection volume of 5 – 10 µL (~20 µg of protein at ~1 mg/mL [5 µM]). Positive Ion Electrospray ionization mode on a Waters Micromass ® Q-Tof micro ™ was in operation. Data processing was performed using Advion 'Chipsoft' software.

Supplementary References

Gherzi, R. et al. DIRECT MODULATION OF INSULIN-RECEPTOR PROTEIN TYROSINE KINASE BY VANADATE AND ANTI-INSULIN RECEPTOR MONOCLONAL-ANTIBODIES. Biochemical and Biophysical Research Communications 152, 1474-1480, doi:10.1016/s0006-291x(88)80452-2 (1988).

Posner, B. I. et al. PEROXOVANADIUM COMPOUNDS - A NEW CLASS OF POTENT PHOSPHOTYROSINE PHOSPHATASE INHIBITORS WHICH ARE INSULIN MIMETICS. Journal of Biological Chemistry 269, 4596-4604 (1994).

Supplementary figure legends

Supplementary Figure S1. (A) SEC molecular weight standards as determined on a Superdex 200 column. (B) Elution profile of the GyrB-FGFR1KD.TC monomer as determined on a Superdex 200 column.

Supplementary Figure S2. Specific and quantitative labelling of GyrB.FGFR1KD.TC upon incubation with FlAsH-EDT_2 demonstrated by SDS-PAGE (Coomassie staining and in-gel fluorescence).

Supplementary Figure S3. (A) (B) Inhibitor dose response titration of GyrB.FGFR1KD.TC in the presence of 50 µM ATP, 0.2 µg/µl PolyE_4Y_1 peptide and inhibitor PD173074 at varying concentrations of 0.8 – 500 nM (FGFR1 IC_{50} = ~22 nM). Kinase inhibition was measured by detecting the decrease in phosphorylation of PolyE_4Y_1 after 1 hour. Maximal activity of GyrB.FGFR1KD.TC and background signal were measured in the absence of inhibitor PD173074.

Supplementary Figure S4. Complex formation of FGFR1KD with mTFP.nSH2 and artificial dimerization as observed by chromatography

(A) Complex formation; Elution profiles of the GyrB.FGFR1KD.TC-mTFP.nSH2 complex (blue) and the excess nSH2 (pink) with 50 µM ATP/20 mM $MgCl_2$ as determined on a Superdex 200 column.

(B) Elution profiles of the GyrB.FGFR1KD.TC dimer (green) and the GyrB.FGFR1KD.TC monomer (yellow) with 125 µM coumermycin as determined on a Superdex 200 column.

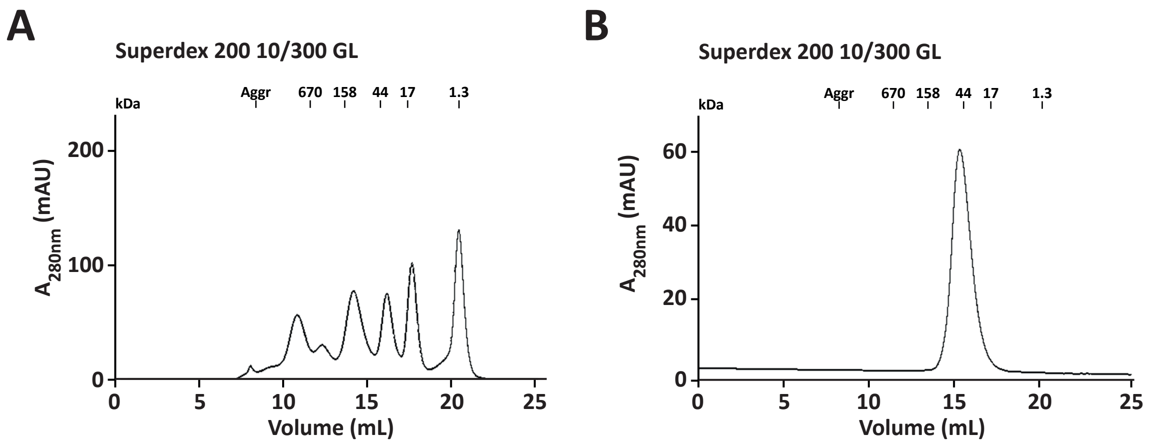

Supplementary Figure S1

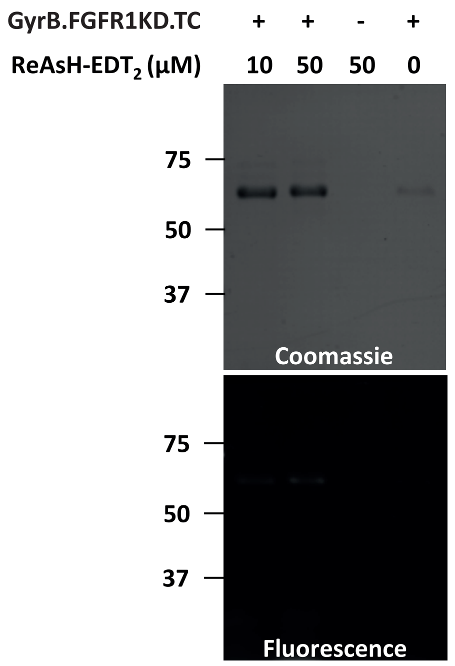

Supplementary Figure S2

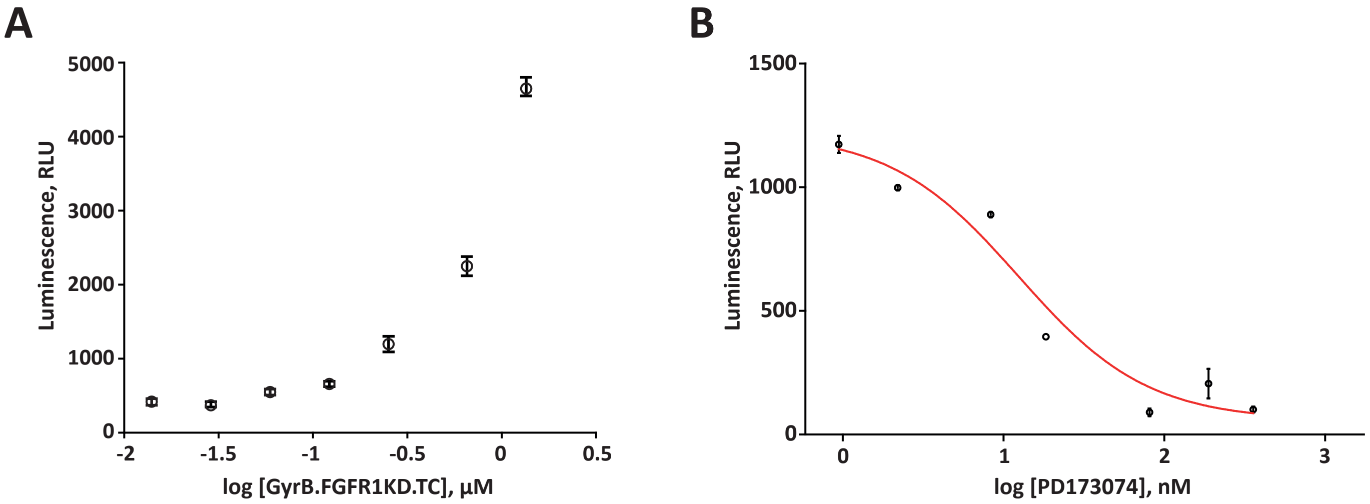

Supplementary Figure S3

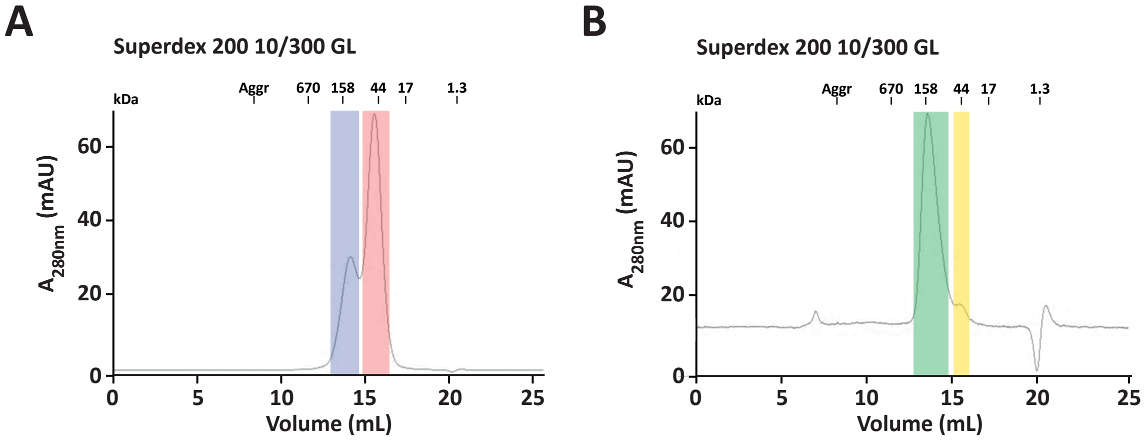

Supplementary Figure S4
